# Fit-for-Purpose Assessment of QuEChERS LC-MS/MS Methods for Environmental Monitoring of Organotin Compounds in the Bottom Sediments of the Odra River Estuary

**DOI:** 10.3390/molecules27154847

**Published:** 2022-07-28

**Authors:** Dawid Kucharski, Robert Stasiuk, Przemysław Drzewicz, Artur Skowronek, Agnieszka Strzelecka, Kamila Mianowicz, Joanna Giebułtowicz

**Affiliations:** 1 Department of Bioanalysis and Drugs Analysis, Faculty of Pharmacy, Medical University of Warsaw, Banacha 1, 02-097 Warsaw, Poland; jgiebultowicz@wum.edu.pl; 2 Department of Geomicrobiology, Institute of Microbiology, Faculty of Biology, University of Warsaw, Miecznikowa 1, 02-096 Warsaw, Poland; r.stasiuk@biol.uw.edu.pl; 3 Polish Geological Institute-National Research Institute, Rakowiecka 4, 00-975 Warsaw, Poland; przemyslaw.drzewicz@pgi.gov.pl; 4 Institute of Marine and Environmental Sciences, University of Szczecin, Mickiewicza 16, 70-383 Szczecin, Poland; artur.skowronek@usz.edu.pl (A.S.); agnieszka.strzelecka@usz.edu.pl (A.S.); 5 Interoceanmetal Joint Organization, Cyryla i Metodego 9, 71-541 Szczecin, Poland; k.mianowicz@iom.gov.pl

**Keywords:** bottom sediments, environmental monitoring, Odra River estuary, organotin compounds, trueness, verification

## Abstract

Organotin compounds (OTCs) are among the most hazardous substances found in the marine environment and can be determined by either the ISO 23161 method based on extraction with non-polar organic solvents and gas chromatography analysis or by the recently developed QuEChERS method coupled to liquid chromatography-mass spectrometry (LC-MS/MS). To date, the QuEChERS LC/MS and ISO 23161 methods have not been compared in terms of their fit-for-purpose and reliability in the determination of OTCs in bottom sediments. In the case of ISO 23161, due to a large number of interferences gas chromatography-mass spectrometry was not suitable for the determination of OTCs contrary to more selective determination by gas chromatography with an atomic emission detector. Moreover, it has been found that the derivatization of OTCs to volatile compounds, which required prior gas chromatography determination, was strongly affected by the sediments’ matrices. As a result, a large amount of reagent was needed for the complete derivatization of the compounds. Contrary to ISO 23161, the QuEChERS LC-MS/MS method did not require the derivatization of OTC and is less prone to interferences. Highly volatile and toxic solvents were not used in the QuEChERS LC-MS/MS method. This makes the method more environmentally friendly according to the principles of green analytical chemistry. QuEChERS LC-MS/MS is suitable for fast and reliable environmental monitoring of OTCs in bottom sediments from the Odra River estuary. However, determination of di- and monobutyltin by the QuEChERS LC-MS/MS method was not possible due to the constraints of the chromatographic system. Hence, further development of this method is needed for monitoring di- and monobutyltin in bottom sediments.

## 1. Introduction

Organotin compounds (OTCs) belong to the most hazardous substances found in marine environments. The most common OTCs include tributyltin (TBT), triphenyltin (TPhT), monobutyltin (MBT), and dibutyltin (DBT). MBT and DBT are degradation products of TBT [1]. The main sources of TBT and TPhT contamination are antifouling paints that are widely used in the marine industry for the protection of ship hulls and marine structures as well as pesticides used for the preservation of wood, paper, textiles, leather, and plastics. Although the application of OTCs in antifouling coatings has been banned since 2008, the compounds are still present in the environment due to their wide industrial applications (especially in the production of polyurethane foam and silicons) [2,3]. OTCs are introduced into the environment by effluents from wastewater treatment plants. Moreover, the results of many studies indicated the historical contamination of sediments in coastal areas of Poland, especially in the vicinity of harbors and shipyards [4,5,6].

Due to the lipophilic properties of OTCs (logP ≈ 4), routine extraction of organotin compounds from soils, sediments, and sludges is conducted by the use of non-polar organic solvents such as hexane or dichloromethane, frequently with a complexing agent such as tropolone or polycarboxylic acids [7,8]. Recently, to improve extraction efficiency, several sample preparation methods have been developed such as accelerated solvent extraction, microwave extraction, liquid-phase microextraction, solid-phase extraction, and solid-phase microextraction [9]. Those methods allow one to reduce solvent consumption, shorten preparation time, improve recovery, and decrease the limits of detection. The ultrasound-assisted solvent extraction of OTCs from bottom sediments is a well-known ISO standard method [10].

The isolated OTCs are mainly analyzed by gas chromatography (GC) coupled with mass spectrometry (MS), flame photometric detectors, atomic emission detectors (AED), or inductively coupled plasma mass spectrometry [9]. However, OTCs require the derivatization of volatile compounds before their determination by GC-based methods. The most common derivatization procedures include conversion with alkyloborates (e.g., NaBEt_4_), Grignard reagents, or borohydride species (e.g., sodium borohydride—NaBH_4_). Regardless of the particular derivatization method, this step is prone to error, tedious, and laborious. Additionally, very toxic and expensive chemical agents are used during the derivatization procedure. As a result, the methods based on derivatization do not follow the rules of green analytical chemistry (GAC). According to GAC rules, the amount of chemicals and samples consumed during an analytical procedure is strongly restricted. Thus, analytical methods without the derivatization step are preferred [11,12]. The recently developed QuEChERS extraction method combined with liquid chromatography coupled with tandem mass spectrometry for the determination of OTCs in bottom sediments meets GAC rules [6,13]. In comparison to the standard method ISO 23161:2018, QuEChERS extraction requires a smaller amount of solvent and chemical agents as well as a lower mass portion of a sample. Extraction of OTCs by QuEChERS also takes less time than other methods.

Although concentrations of OTCs in sediments are generally higher than in the water column [14], the determination of OTCs is very difficult. Bottom sediments contain a large number of compounds, in many cases with high molecular mass that are extracted together with OTCs. As a result, a lower recovery, reliability, and accuracy of the method are frequently observed. Moreover, the derivatization of OTCs to volatile compounds is very ineffective due to the decomposition of the derivatization reagent or its side reactions with co-extracted matrix constituents [15]. Thus, a comprehensive comparison and fit-for-purpose assessment of two analytical methods for the determination of OTCs in bottom sediments is the subject of this study. The QuEChERS extraction method combined with LC-MS/MS is compared with the solvent extraction method combined with GC-MS and GC with atomic emission detector (AED) [10]. Additionally, BCR 646, a reference material, as well as 10 sediments collected from the Odra River estuary area, SW Baltic Sea, were used for testing the comparability and fit-for-purpose assessment of the aforementioned methods.

## 2. Results and Discussion

The subject of the study was the comparison of the standard method ISO 23161 for the determination of OTCs in bottom sediments with the newly developed QuEChERS LC-MS/MS method. This is a continuation of our earlier studies on the application of the QuEChERS method for OTCs determination [6]. Detailed comparisons of extraction procedures and separation parameters are presented in Tables S1 and S2. In the case of ISO 23161, acid extraction and subsequent derivatization with tetraethylborate are applied in the determination of tin compounds. This method required a larger amount of sediment (at least 1 g d.w.), whereas, in the case of QuEChERS extraction, 125 mg d.w. of the sample amount was enough for the analysis. Analyzing organic tin compounds according to the ISO method requires also a higher amount of reagents. Thus, the cost of the single analysis seems to be higher in comparison to the QuEChERS LC-MS/MS method. Additionally, hexane as the analyte extractant is used in the ISO method. The application of hexane in analytical chemistry is strongly limited by occupational and health safety regulations due to its high neurotoxicity and detrimental effect on lab workers as well as the environment [16]. Contrary to ISO 23161, acetonitrile with 5% formic acid as the organic phase is used in the QuEChERS method. Although acetonitrile is a hazardous reagent, it is generally less toxic than hexane due to its low volatility. According to the Pfizer company’s solvent selection guidelines, the application of acetonitrile in analytical procedures is preferable due to lower toxicity and environmental risk in comparison to other solvents [17]. However, the main disadvantage of the ISO method is the derivatization of OTCs to volatile compounds before their determination by GC. Derivatization of OTCs with sodium tetraethylborate (NaBEt_4_) in the presence of other compounds extracted from complex matrices, such as sediments, requires a large volume of very expensive and toxic reagents. Due to the decomposition of derivatization reagent or the side reactions with other matrix constituents, only part of OTCs may undergo derivatization to volatile compounds [18].

### 2.1. Fit-for-Purpose of the Methods

**Figure 1:**
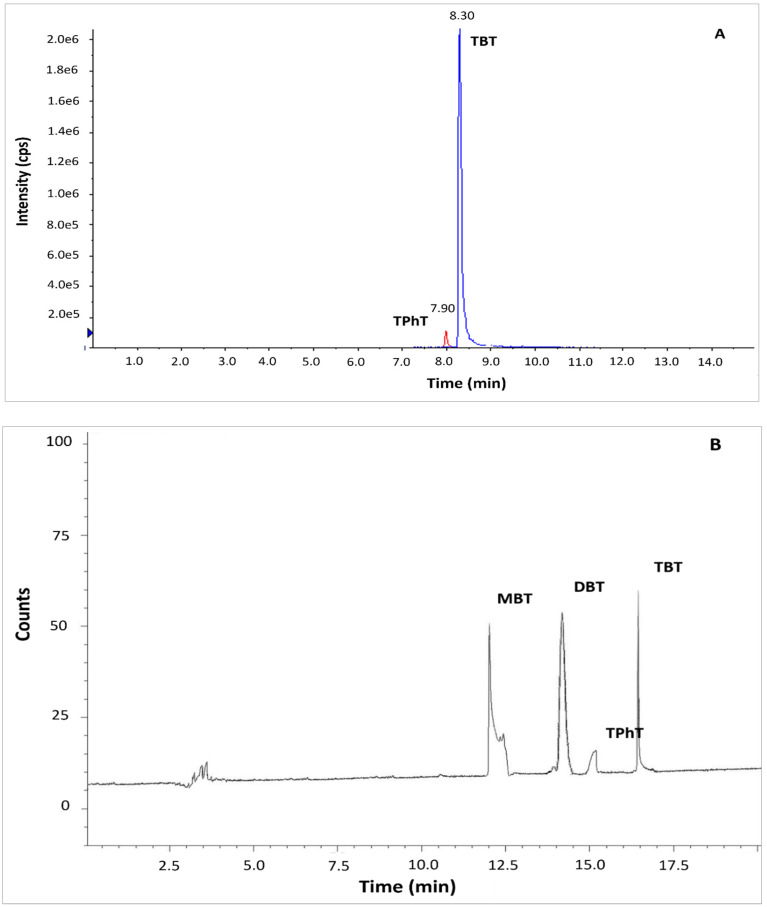
Chromatograms of organotin compounds determined in BCR sediments by (**A**) QuEChERS coupled to LC-MS/MS (**B**) ISO 23161 GC-AED method. Determination of DBT and MBT was only possible using the method ISO 23161 GC-AED. Abbreviations: MBT, monobutyltin; DBT, dibutyltin; TBT, tributyltin; TPhT, triphenyltin.

The standard method ISO 23161 and QuEChERS LC-MS/MS method were tested based on reference material BCR 646 and sediments collected from the Odra River estuary. In the case of standard method ISO 23161, co-extracted matrix constituents strongly interfered in the determination of OTCs by GC-MS. Application of GC-MS required additional clean-up of the solvent extract before derivatization of OTCs to the volatile compounds. The clean-up step is a source of additional bias in the method. It is also very tedious and time-consuming. The application of a very selective GC-AED method allowed for the precise and accurate determination of OTCs in bottom sediments without the clean-up step (Figure 1B). Application QuEChERS LC-MS/MS method resulted in a sufficient separation. The peaks of TBT and TPhT were identified and integrated easily (Figure 1A). Although MBT and DBT are quantitatively extracted by the QuEChERS method, the compounds were not detected by LC-MS/MS due to the constraints of the chromatographic system. The application of ion trap-mass spectrometry or ICP –MS as a detector, addition of tropolone to the eluent, or application of another stationary phase in the LC separation column may solve the problem [19,20,21].

Due to the necessity of solvent exchange, the QuEChERS extraction method is not recommended for sample preparation before determination by gas chromatography. Therefore, QuEChERS LC-MS/MS and standard method ISO 23161 combined with GC-AED were taken for further comparison.

### 2.2. Verification of the Methods 

Analytical methods ISO 23161 GC-AED and QuEChERS LC-MS/MS were verified according to Eurachem Guide [22] in terms of limit of detection (LOD), the limit of quantification (LOQ), linearity, accuracy, precision, and recovery (Table 1). The LOQ and LOD values were similar for both methods. In the case of QuEChERS LC-MS/MS, higher LOD values were observed for TPhT (5.0 ng·g^−1^ d.w.) due to the high signal-to-noise ratio. The coefficients of determination (R^2^) of the calibration curves were ≥0.99. The accuracy did not meet the acceptance criteria (85–115%) for MBT in ISO 23161GC-AED and was in the range of 80–85%. The precision for MBT in ISO 23161-GC-AED did not meet the acceptance criteria (≤15%) and was 27%. For the other OTCs, the precision was lower than 15%. The recovery in most cases was higher than 85%. For the ISO 23161 GC-AED method, MBT recovery was lower than 80%. 

The trueness of both methods was also tested. The differences between OTCs concentration in the dry weight of the sample assigned to the certified reference and the mean of concertation determined by the QuEChERS LC-MS/MS method were as follow: B_TBT_ = 30.0 ng·g^−1^ d.w. and B_TPhT_ = 1.1 ng·g^−1^ d.w. The concentration determined by ISO 23161-GC-AED were as follow B_TBT_ = 47.8 ng·g^−1^ d.w., B_TPhT_ = 1.2 ng·g^−1^ d.w., B_DBT_ = 68.8 ng·g^−1^ d.w., and B_MBT_ = 103.9 ng·g^−1^ d.w. The differences between determined and reference concentrations of OTC in the material were within *U*_Δ_ (calculating according to Equation (1)). Thus, the determined concentrations were compatible with reference concentrations.

### 2.3. Analytical Method Comparison Based on the Real Samples of Bottom Sediments

**Figure 2:**
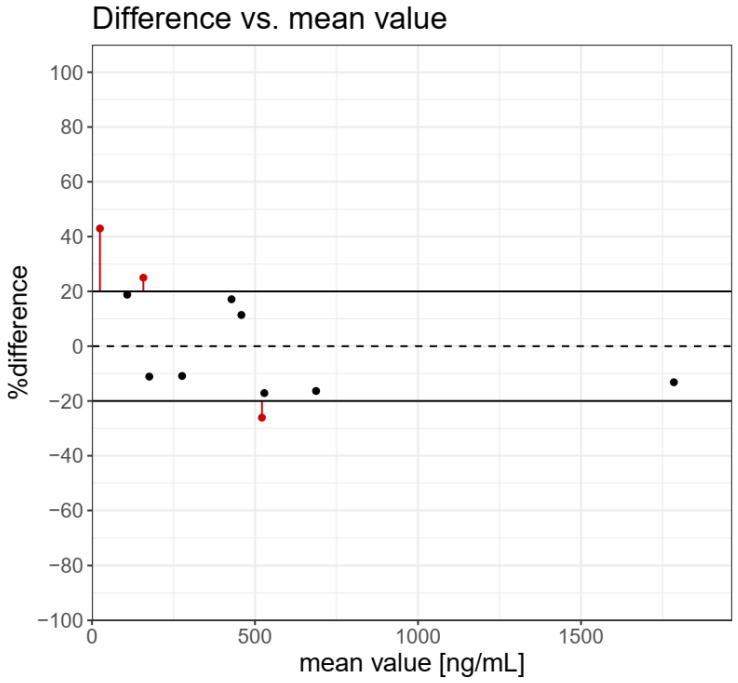
Difference vs. mean value for tributyltin determination in real sediments samples and BCR 464 freshwater sediment. The cumulative %ISR for 11 samples was 72.7% and met acceptance criteria (≥67%) for comparability of the analytical methods tested.

The results of the determination of TBT, TPhT in BCR-646 freshwater sediments, and 10 samples of sediment from the Odra river estuary by the use of QuEChERS-LC-MS/MS and ISO 23161 GC-AED methods are presented in Table 2. Incurred Sample Reanalysis (ISR) tool was used to the assessment of the reproducibility of analytical methods [23]. The results for TBT determination in reference and real samples of sediments are presented in Figure 2. The graph presents individual ISR data points and concentration-dependent trends. If the difference between the concentrations determined by the use of the two methods is within 20% of the mean from at least 67% of the measurements, then the two methods are considered comparable. A difference higher than 20% was observed for three samples in low and medium concentrations. The cumulative %ISR calculated for 11 samples was 72.7% and met acceptance criteria (≥67%) for the comparability of the tested analytical methods.

Based on the Wilcoxon test of six replicates of freshwater sediment BCR-464 analyses, it was confirmed that the results of determination of TBT and TPhT by the use of these methods were not statistically different. In the case of TBT determination, the result of the test was *p =* 0.0809 whereas, in the case of TPhT, it was *p =* 0.6625. In real samples of bottom sediments from the Odra River estuary, TBT concentrations in dry weight of sample determined by QuEChERS LC-MS/MS varied from 29.4 to 1667.5 ng·g^−1^, whereas, in the case of ISO 23161 GC-AED, the determined concentrations varied from 19.0 to 1862.5 ng·g^−1^. Based on the Wilcoxon signed-rank test, it has been found that the results of TBT determination by the use of these methods were not statistically different (*p =* 0.91). TPhT was found only in the two samples of bottom sediments (samples #7 and #8). The concentration was determined both by the use of QuEChERS LC-MS/MS and ISO 23161 GC-AED (Table 2). 

MBT and DBT at dry weight concentration levels above the LOQ were found in samples #7 (19.6 and 177.5 ng·g^−1^.) and #8 (17.3 and 522.4 ng·g^−1^), respectively (Table 2). The concentrations of these compounds in the Szczecin Lagoon (sample #3) were close to those reported in 2008 [5]. The highest DBT and MBT concentrations in the dry weight of the sample were 76.0 ng·g^−1^ and 67.0 ng·g^−1^. In this study, determined DBT and MBT concentrations in the dry weight of the sample were 62.3 and 23.9 ng·g^−1^, respectively. In 2008, it was reported that TPhT has not been found in sediment samples from the Szczecin Lagoon [5]. 

In the Szczecin Lagoon, the dry weight concentration of TBT reported in 2008 was 453.2 ng·g^−1^ [5], whereas, in this study, the concentration was 283.1 ng·g^−1^. The occurrence of OTCs in the Szczecin Lagoon was also reported in 2018 [24]. The highest concentrations were 152.9 ng·g^−1^ for TBT, 66.1 ng·g^−1^ for DBT, and 67.1 ng·g^−1^ for MBT. OTCs concentrations in the sediment collected in the vicinity of the Szczecin harbor (sample #8) were 1667.5 ng·g^−1^ for TBT, 231.6 ng·g^−1^ for DBT, and 156.7 6 ng·g^−1^ MBT. Comparatively, in the sediment collected in Gdynia harbor, the mean dry weight concentrations were 2148.2 ng·g^−1^ for TBT, 751.9 ng·g^−1^ for DBT, and 261.4 ng·g^−1^ for MBT [25]. This indicates that the cleaning of ship surfaces by sandblasting is a source of OTCs pollution. Moreover, OTCs are persistent in bottom sediments. Thus, bottom sediments may be a potential source of environmental pollution by OTCs. 

## 3. Materials and Methods

### 3.1. Experimental

Pure standards of tributyltin chloride (TBT, 96%), triphenyltin chloride (TPhT, 95%), dibutyltin chloride (DBT, 96%), monobutyltin (MBT, 95%) and internal standard, deuterated tributyltin chloride-d27 (TBT-d27, 96%) were purchased from Sigma Aldrich (St. Louis, MO, USA). HPLC gradient-grade methanol, acetonitrile, and formic acid 98% were purchased from Merck (Darmstadt, Germany). QuEChERS salts: magnesium sulfate, sodium chloride, sodium acetate, and ammonium acetate were purchased from Chempur (Piekary Śląskie, Poland), whereas trisodium citrate from Avantor (Gliwice, Poland). Acetic acid, methanol, hexane, and tetrahydrofurane of GC purity were purchased from Sigma Aldrich (St. Louis, MO, USA). Sodium tetraethylborate purity was 97% from Sigma Aldrich (St. Louis, MO, USA) and disodium citrate was bought from Acros Organics (Morris Plains, NJ, USA). Certificated Reference Material BCR^®^ 646 was purchased from Merck (Darmstadt Germany). Ultrapure water was obtained from a Millipore water purification system (MiliQ, Billerica, MA, USA) equipped with a UV-lamp (resistivity of 18.2 MΩ.cm (at 25 °C) and a TOC value below 5 ppb). 

The stock solutions of 1 mg∙mL^−1^ of TBT, TPhT, DBT, MBT, and TBT-d27 were prepared in methanol. The working standard solutions were prepared by dilution of the stock solution with an appropriate amount of methanol just before use. All stock solutions were stored at −25 °C.

### 3.2. Organotin Compounds Extraction

#### 3.2.1. Extraction of Organotin Compounds for LC-MS/MS Analysis (QuEChERS Extraction Procedure)

A dry sample of sediment was weighed (0.125 g) and placed in 2 mL Eppendorf^®^ tubes. As an internal standard, 25 µL of deuterated tributyltin (TBT-d27) was added, followed by 200 µL of MiliQ water. Then, the samples were vigorously shaken by the use of a vortex shaker for 1 min and 250 µL of 5% formic acid in acetonitrile was added. The samples were put in a vessel with ice and 100 mg of ammonium acetate was added. Then they were extracted by ultrasonication for 5 min and shaken for 15 min (1500 rpm). The samples were centrifuged for 5 min (relative centrifuge force was 4472× *g*) and the supernatant was collected for further analysis.

#### 3.2.2. Extraction of Organotin Compounds for GC Analysis (ISO 23161 Extraction Procedure)

Extraction of OTCs from dry solid samples was carried out according to ISO 23161. Firstly, 3.2 g of lyophilized sediment was ultrasound extracted for 30 min. with 3 mL of the mixture of acetic acid:methanol: water (1:1:1; *v/v/v*). The extraction procedure was repeated with 1.5 mL of the mixture, and liquid phases were combined. Next, the pH was adjusted to 4.5 with acetic acid, and 5 mL of hexane and 10% sodium tetraethylborate in tetrahydrofurane (0.5 mL per gram of solid sample) was added. After that, the organic phase was concentrated to 1 mL.

### 3.3. Instrumental Methods

#### 3.3.1. LC-MS/MS

Liquid chromatography with tandem mass spectrometry (LC-MS/MS) analysis was performed using Agilent 1260 Infinity (Agilent Technologies, Santa Clara, CA, USA) equipped with a degasser, autosampler, and binary pump, coupled to a Hybrid Triple Quadrupole/Linear Ion trap mass spectrometer (QTRAP^®^ 4000, AB SCIEX, Framingham, MA, USA). The curtain gas, ion source gas 1, ion source gas 2, and collision gas (all high purity nitrogen) were set at 280 kPa, 380 kPa, 410 kPa, and “high” instrument units, respectively. The ion spray voltage and source temperatures were 5500 V and 600 °C, respectively. Kinetex RP-18 column (100 mm, 4.6 mm, particle size 2.6 µm) supplied by Phenomenex (Torrance, CA, USA) was used. The column temperature was 40 °C; the eluent flow rate was 0.5 mL/min. The eluent was prepared from two solutions: A-0.2% formic acid in water and B-0.2% formic acid in acetonitrile. The concentration of solution B in the eluent was 5% for 2 min, after that, the concentration increased to 95% in 7.5 min and for the next 5 min was 95%. The injection volume was 10 µL. The organotin compounds were analyzed in multiple reaction monitoring (MRM) mode. Two ion transitions (precursor ion and fragment ion) were *m/z* 291 → 179 for TBT, 351 → 196 for TPhT, and 318 → 190 for d-TBT. 

#### 3.3.2. GC-MS

The separation of organotin compounds was performed using an Agilent 7890A Series Gas Chromatograph interfaced to an Agilent 5973c Network Mass Selective Detector and an Agilent 7683 Series Injector (Agilent Technologies, Santa Clara, CA, USA). A 5 μL sample was injected in splitless mode (volume relative standard deviation was 0.3%) to an HP-5MS column (30 m × 0.25 mm I.D., 0.25 μm film thickness, Agilent Technologies, Santa Clara, CA, USA) using helium as the carrier gas at 1 mL/min flow. The ion source was maintained at 230 °C; the GC oven was programmed with a temperature gradient starting at 40 °C for 3 min, then increased with a 10 °C/min rate to 220 °C, held for 5 min, after that increased to 20 °C/min rate to 300 °C, and held for 10 min. MS was carried out in the electron-impact mode at an ionizing potential of 70 eV. Mass spectra were recorded in the range of 40–800 mass-to-charge ratio (*m/z*). Identification of the selected organic compounds was performed with an Agilent Technologies Enhanced ChemStation (G1701EA ver. E.02.00.493) and The Wiley Registry of Mass Spectral Data (version 3.2, Copyright 1988–2000 by Palisade Corporation with, 8th 213 Edition with Structures, Copyright 2000 by John Wiley and Sons, Inc., Hoboken, NJ, USA) using a 3% cut-off threshold.

#### 3.3.3. GC-AED

Organotin compounds were determined by GC (GC 7890A, Agilent Technologies, USA) coupled with atomic emission detector (AED) model JAS G2350A from Joint Analytical System (Moers, Germany). The samples (5 μL) were manually injected by the use of a 10 μL syringe. The injector was in splitless mode and kept at 280 °C. The organic compounds were separated using an HP-5 column (30 m, 0.32 mm I.D., 0.25 μm particle size, Agilent Technologies, Santa Clara, CA, USA) using helium as the carrier gas (1 mL/min). The temperature gradient was as follows: 40 °C for 3 min, then increased with 10 °C/min rate to 220 °C, held for 5 min, then increased with 20 °C/min rate to 300 °C, and after that, held for 10 min. Helium plasma was used as an excitation source in the AED detector. Detection lines for Sn were 301 and 303 nm. The temperature of the transfer line was 280 °C. The flows of the remaining reaction gases (oxygen, nitrogen, hydrogen) used in the determination of tin were set according to the manufacturer’s recommendations. Software from Joint Analytical System, Moers, Germany (version D.02.01 was used for the calculation of OTCs concentrations based on the peak area of the analyte. All determinations were made in triplicate and averaged.

### 3.4. Verification Procedure

Verification of the analytical methods was performed in terms of limits of quantification (LOQ), limits of detection (LOD), analytical ranges (linearity), accuracy, precision, and recovery. The limit of detection (LOD) was estimated by determining the S/N of the minimum measured concentrations and extrapolating to the S/N value equals 3. Limit of quantification (LOQ) was established as the concentration of OTCs, for which MS signal-to-noise ratio is equal to or greater than 10, with precision below 20% and accuracy ± 20%. Linearity was evaluated based on a relation between peak area ratio (or peak area) and analyte concentration that can be represented as a straight line. A linear regression method was used to test linearity. Calibration curves were prepared in triplicate. The precision and accuracy of the analytical methods were determined based on six repetitions. Recoveries were determined based on six repetitions, comparing peak areas of target analytes extracted from blank samples to the peak areas of standard solution. The certified reference material BCR 646 (European Commission Joint Research Centre Institute for Reference Materials and Measurements, Geel, Belgium) was used to evaluate the accuracy, precision, and trueness of the results. The material consists of a dried and ground freshwater bottom sediment with a particle size (<90 µm), reference concentrations of OTCs with their uncertainty ($U_{\Delta }$) were as follows: 480 ng·g^−1^ ($U_{\Delta }=$ 80 ng·g^−1^) for TBT, 770 ng·g^−1^ ($U_{\Delta }$ = 90 ng·g^−1^) for DBT, 610 ng·g^−1^ for MBT ($U_{\Delta }$ = 120 ng·g^−1^) and 29 ng·g^−1^ ($U_{\Delta }$ = 11 ng·g^−1^) for TPhT. Trueness is typically determined in terms of bias (B) by comparison of the obtained results with the reference value. Significance testing of the bias that took into account uncertainty of certificated value was performed. According to ISO Guide 33 [26] (ISO, 2015), the expend uncertainty of the difference between certificated and measured value *U*_Δ_ with coverage factor *k* = 2, corresponding to a level of confidence of 95%, is obtained by Equation (1):

(1) $$U_{\Delta }=k\cdot \sqrt{u_{ref}^{2}+\frac{S_{m}^{2}}{n}}$$

where: *u_ref_* is the uncertainty of the reference value taken from the certificate (40 ng·g^−1^ for TBT and 5.5 ng·g^−1^ for TPhT).

*s_m_* is a standard deviation calculated from the measured values.
*n* is several repeated measurements, (*n* = 6).

### 3.5. Statistical Methods

A comparison of the analytical methods was performed by the use of Incurred Sample Reanalysis (ISR) tool developed by Rudzki et al. [23] and the Wilcoxon signed-rank test. For ISR the %difference with fixed acceptance limits was set at −20 and 20% and the %ISR should be at least 67% to confirm the equivalence of the methods. For the Wilcoxon signed-rank test, a *p*-value of 0.05 or less was considered significant. The statistical analysis of the results was performed with the STATISTICA version 13.1 for Windows (TIBCO Software Inc., Palo Alto, CA, USA)

### 3.6. Characteristics of Bottom Sediment Samples

Sediment samples were collected from the Odra River estuary by the use of the Van Veen grab sampler, collecting surface sediments up to 20 cm deep. Samples were kept at 4 °C till their delivery to the laboratory. Then, they were frozen at −80 °C, freeze-dried, and stored at −80 °C till analysis. Before analysis, the sediments were grounded in an agate mortar and sieved through a 0.063 mm sieve. Geographical localization of sampling sites and depths of sampling are presented in Table 3.

The relevant physicochemical parameters (total organic carbon content (TOC), total nitrogen content (N), acid volatile sulfur content (AVS), total phosphorus content (P), and granulometric composition (% of silt fraction and % of clay fraction)) are presented in Table S3 in Supplementary materials. Elemental analysis (N, P), TOC, and AVS analyzes were performed at the Polish Geological Institute-National Research Institute by the use of analytical methods accredited according to ISO-17025 standard. Granulometric analysis was performed at the University of Szczecin (according to the ISO 13320 standard method).

## 4. Conclusions

The newly developed QuEChERS LC-MS/MS method for the determination of OTCs in bottom sediments was compared with both GC-MS and GC-AED based standard method ISO-23161. Due to the high number of interferences, the determination of OTCs by GC-MS was very difficult and not recommended for environmental monitoring of bottom sediments. The GC-AED method is more selective and free from interferences. However, the determination of OTCs by QuEChERS LC-MS/MS required less time and it is more convenient for laboratory staff. The method does not require clean-up, derivatization step, and application of very toxic, volatile reagents. Therefore, QuEChERS-LC-MC/MS is more suitable for routine environmental monitoring of OTCs in a large number of bottom sediment samples. The results of OTCs determination in real bottom sediments by QuEChERS-LC-MC/MS and ISO 23161 were compatible. However, the degradation product of TBT, MBT, and DBT could not be detected by LC-MS/MS. The application of other chromatographic settings may likely alleviate the problem of MBT and DBT determination by LC-MS/MS. MBT and DBT were extracted quantitatively by the QuEChERS extraction method. However, the method cannot be applied in the determination of OTC by GC methods due to the necessity of solvent exchange.

## Figures and Tables

**Table 1:** Validation parameters of QuEChERS LC-MS/MS and ISO 23161 GC-AED methods for monobutyltin (MBT), dibutyltin (DBT), tributyltin (TBT), and triphenyltin (TPhT).

Parameters	QuEChERS (LC-MS/MS)	ISO 23161 (GC-AED)
Limit of quantification (LOQ)	2.0 ng·g^−1^ d.w. TBT 5.0 ng·g^−1^ d.w. TPhT	1.7 ng·g^−1^ d.w. MBT 2.4 ng·g^−1^ d.w. DBT 2.5 ng·g^−1^ d.w. TBT 3.0 ng·g^−1^ d.w. TPhT
Limit of detection (LOD)	0.6 ng·g^−1^ d.w. TBT 1.5 ng·g^−1^ d.w. TPhT	0.5 ng·g^−1^ d.w. MBT 0.7 ng·g^−1^ d.w. DBT 0.7 ng·g^−1^ d.w. TBT 0.9 ng·g^−1^ d.w. TPhT
Analytical ranges (linearity)	2.0–4000.0 ng·g^−1^ d.w.; R^2^_TBT_ = 0.99 5.0–4000.0 ng·g^−1^ d.w.; R^2^_TPhT_ = 0.99	1.7–4000.0 ng·g^−1^ d.w; R^2^_MBT_ = 0.99 2.4–4000.0 ng·g^−1^ d.w.; R^2^_DBT_ = 0.99 2.5–4000.0 ng·g^−1^ d.w.; R^2^_TBT_ = 0.99 3.0–4000.0 ng·g^−1^ d.w.; R^2^_TPhT_ = 0.99
Accuracy (*n* = 6) ^a^	98.0–105.0%-TBT 91.0–101.0%-TPhT	80.0–85.0%-MBT 88.0–93.0%-DBT 88.0–92.0%-TBT 87.0–105.0%-TPhT
Precision (*n* = 6) ^b^	3.0%-TBT 4.9%-TPhT	27.0%-MBT 15.0%-DBT 6.0%-TBT 2.0%-TPhT
Recovery (*n* = 6)	93%-TBT 91%-TPhT	79%-MBT 86%-DBT 90%-TBT 91%-TPhT

^a^ acceptance criteria: 85–115%; ^b^ acceptance criteria: ≤15%

**Table 2:** The results of (TBT), triphenyltin (TPhT), dibutyltin (DBT), and monobutyltin (MBT) determination in real sediments samples and reference material of freshwater sediment (BCR 646) by the use QuEChERS LC-MS/MS and ISO GC-AED methods. Certified values of BCR-646: TBT-480 ng·g^−1^ (±80 ng·g^−1^), TPhT-29 ng·g^−1^ (±11 ng·g^−1^), DBT-770 ng·g^−^^1^ (±90 ng·g^−1^), MBT-610 ng·g^−1^ (±120 ng·g^−1^).

	QuEChERS LC-MS/MS ^a^		ISO GC-AED			
Sample	TBT *n* = 3 ng·g^−1^ d.w	TPhT, *n* = 3 ng·g^−1^ d.w	TBT, *n* = 3 ng·g^−1^ d.w	TPhT, *n* = 3 ng·g^−1^ d.w	DBT, *n* = 3 ng·g^−1^ d.w	MBT, *n* = 3 ng·g^−1^ d.w
S1	29.4 ± 1.1	<5.0	19.0 ± 1.1	<3.0	<2.4	<1.7
S2	177.2 ± 11.2	<5.0	137.8 ± 8.3	<3.0	23.4 ± 3.5	<1.7
S3	453.2 ± 7.4	<5.0	589.1 ± 35.4	<3.0	62.3 ± 9.4	23.9 ± 6.5
S4	261.3 ± 12.6	<5.0	291.3 ± 17.5	<3.0	522.4 ± 78.4	177.5 ± 47.9
S5	165.9 ± 19.3	<5.0	185.4 ± 11.1	<3.0	17.3 ± 2.6	52.3 ± 14.1
S6	483.4 ± 11.5	<5.0	573.9 ± 34.4	<3.0	137.4 ± 20.6	19.6 ± 5.3
S7	464.5 ± 22.7	15.1 ± 0.3	391.2 ± 23.5	11.0 ± 0.2	73.8 ± 11.1	46.2 ± 12.5
S8	1667.5 ± 57.8	21.9 ± 0.6	1862.5 ± 111.8	29.9 ± 0.6	231.6 ± 34.8	156.7 ± 42.3
S9	118.1 ± 4.0	<5.0	97.8 ± 5.9	<3.0	58.6 ± 8.8	99.6 ± 26.9
S10	631.1 ± 24.6	<5.0	743.4 ± 44.6	<3.0	<2.4	<1.7
BCR ^a^	484.2 ± 14.4	29.6 ± 1.4	432 ± 6.1	27.8 ± 1.8	698.0 ± 14.2	483.8 ± 24.7

^a^ the analyses of BCR were replicated six times.

**Table 3:** Geographic coordinates, depth of sampling from the water surface, and localization name of sediments collected from the Odra River estuary.

Sample	Geographic Coordinates		Depth of Sampling from the Surface (m)	Localization
	Latitude N	Longitude E		
S1	53°51.209′	014°18.107′	1.5	Karsibór next to Rybaczówka
S2	53°59.570′	014°42.332′	2.1	West Dziwna River
S3	53°41.456′	014°25.297′	4.4	Szczecin Lagoon-Brzózki
S4	53°38.538′	014°35.958′	3.5	Roztoka Odrzańska-near to Stępnicka Bay
S5	53°33.572′	014°34.774′	1.8	Police-Larpia
S6	53°27.621′	014°36.102′	2.2	Sailing Canal, near Święta
S7	53°27.336′	014°35.434′	2.5	West Odra River-Gryfia Shipyard Dock No. 5
S8	53°27.328′	014°35.968′	2.2	Szczecin: opposite Gryfia Island
S9	53°26.300′	014°35.280′	10.3	Elevator “Ewa”
S10	53°23.827′	014°38.205′	3.6	Dąbie Marina

## Data Availability

Not applicable.

## Supplementary Materials

The following supporting information can be downloaded at: https://www.mdpi.com/article/10.3390/molecules27154847/s1. Table S1: Parameters of QuEChERS and ISO 23161 extraction procedures; Table S2: Separation and detection parameters of LC-MS/MS, GC-AED, and GC-MS techniques; Table S3: Granulometric composition (% of silt fraction and % of clay fraction) and content in mg·kg-1 of total organic carbon (TOC), total nitrogen content (N), acid volatile sulfur (AVS) and, total phosphorus (P) of sediments collected from the Odra River estuary.

## Sample Availability

Samples of the compounds are not available from the authors.
