# Supplementary material for: Fit-for-Purpose Assessment of QuEChERS LC-MS/MS Methods for Environmental Monitoring of Organotin Compounds in the Bottom Sediments of the Odra River Estuary
Source: Molecules. 2022 Jul 28;27(15):4847. doi: 10.3390/molecules27154847 (PMC9370016; doi:10.3390/molecules27154847)
Supplement: Supplementary file 1 [file molecules-27-04847-s001.zip › molecules-1814849-supplementary.pdf]

Fit-for-Purpose Assessment of QuEChERS LC-MS/MS Methods for Environmental Monitoring of Organotin Compounds in the Bottom Sediments of the Odra River Estuary

Table S1. Parameters of QuEChERS and ISO 23161 extraction procedures.

Parameters	QuEChERS	ISO 23161
Sample mass [g]	0.125	1-5
Initial solution	Water	Acetic acid:methanol:water (1:1:1; v/v/v)
The volume of initial solution [ml]	0.2	9
Extractant solution	ACN + 5% formic acid	Hexane +10% sodium tetraethylborate in tetrahydrofurane
Volume of extractant solution [ml]	0.25	5
Internal standard	TBT-d27	TBT-d27
Extraction procedure	1st step- 5 minutes of ultrasound extraction<br>2nd step- 15 minutes shaking (1500 rpm)	1st step - 30 minutes ultrasound extraction with 3 extractants - three times<br>2nd step - Collection of the liquid phase<br>3rd step - Adjustment the pH of the collected liquid phase to 4.5<br>4th step - Shaking the liquid phase with the organic phase twice for 20 minutes (150 rpm)<br>5th step - Evaporation of the collected organic phase to 1 ml

Tabel S2. Separation and detection parameters of LC-MS/MS, GC-AED, and, GC-MS techniques.

Parameters	QuEChERS-LC-MS/MS	ISO GC-AED
Chromatographic separation	Liquid chromatograph Agilent 1260 Infinity (Agilent Technologies, Santa Clara, CA, US)	Gas chromatograph GC 7890A (Agilent Technologies, USA)
Chromatography column	Kinetex RP-18 column (100 mm, 4.6 mm, particle size 2.6 μm) supplied by Phenomenex (Torrance, CA, USA)	HP-5MS column (30m, 0.25 mm I.D., 0.25 μm particle size (Agilent Technologies, USA)
Separation parameters	Column temperature- 40°C;<br>Eluent flow rate- 0.5 ml/min.<br>The eluent was prepared from two solutions: A- 0.2% formic acid in water and B- 0.2% formic acid in acetonitrile. The concentration of solution B in the eluent was 5% for 2 minutes, after that, the concentration increased to 95% in 7.5 minutes and for the next 5 minutes was 95%. The injection volume was 10 μl.	Injection: splitless for 60 s, injection volume: 5 μl<br>Iniection temperature: 280 °C<br>Carrier: helium (1 ml/min) constant flow<br>The temperature gradient of the column: 40°C for 3 min, then increase to 220°C with 10°C/min rate, held for 5 min, then increase to 300 °C with 20°C/min rate, and held for 10 min
Detector	Triple Quadrupole/Linear Ion trap mass spectrometer (QTRAP® 4000, AB SIEX, Framingham, MA, US)	Atomic emission spectrometric detector: AED JAS G2350A (JAS, Germany)

Detection parameters	The curtain gas, ion source gas 1, ion source gas 2, and collision gas (all high purity nitrogen) were set at 280 kPa, 380 kPa, 410 kPa, and "high" instrument units, respectively. The ion spray voltage and source temperatures were 5500 V and 600°C, respectively.	Mass spectrometer: Agilent 5973c Network Mass Selective Detector, (Agilent Technologies, USA)<br>Atomic emission spectrometric detector:<br>Transfer line temperature: 280°C<br>Cavity temperature: 280°C<br>The line for Sn: 301nm, 303 nm<br>Gases: hydrogen (50 psi), oxygen (14 psi)<br>Helium makes up: 270ml/min<br>Mass spectrometer:<br>MSD transfer line temperature: 300 °C<br>Acquisition mode: SCAN<br>Ionization voltage:70eV<br>Source temperature:230°C<br>Quadrupole temperature 150°C

Table S3. Granulometric composition (% of silt fraction and % of clay fraction) and content in $mg\cdot kg^{-1}$ of total organic carbon (TOC), total nitrogen content (N), acid volatile sulfur (AVS), and total phosphorus (P) of sediments collected from the Odra River estuary.

Sample number	Silt [%]	Clay [%]	TOC [$g\cdot kg^{-1}$]	N [$g\cdot kg^{-1}$]	AVS [$g\cdot kg^{-1}$]	P [$g\cdot kg^{-1}$]
S1	97.2	2.8	13.6	3.5	1.7	0.34
S2	96.7	3.3	115.0	14.0	11.7	1.04
S3	97.3	2.7	92.7	10.5	8.2	1.15
S4	96.6	3.4	97.3	9.1	5.6	2.02
S5	96.5	3.5	154	13.7	9.0	2.13
S6	95.5	4.5	88.1	6.6	3.5	2.97
S7	95.7	4.3	70.7	5.3	1.2	2.08
S8	95.5	4.5	75.7	8.2	2.7	3.85
S9	94.6	5.4	24.1	2.4	0.8	1.52
S10	96.2	3.8	91.3	9.1	1.0	1.36
